# Colonic mucosa-associated lymphoid tissue in a renal transplant recipient: a case report

**DOI:** 10.1186/s13256-020-02387-9

**Published:** 2020-06-28

**Authors:** Kylie Martin, Andrew Taylor, Constantine Tam, Prue Hill, David Machet, David Goodman

**Affiliations:** 1 Department of Nephrology, St Vincent’s Hospital Melbourne, 41 Victoria Parade, Fitzroy, Victoria 3065 Australia; 2 Department of Gastroenterology, St Vincent’s Hospital Melbourne, 41 Victoria Parade, Fitzroy, Victoria 3065 Australia; 3 Department of Haematology, St Vincent’s Hospital Melbourne, 41 Victoria Parade, Fitzroy, Victoria 3065 Australia; 4 Department of Anatomical Pathology, St Vincent’s Hospital Melbourne, 41 Victoria Parade, Fitzroy, Victoria 3065 Australia; 5 Anatpath, 120 Gardenvale Road, Gardenvale, Victoria 3065 Australia

**Keywords:** Kidney transplantation, Lymphoma, Immunosuppression, Case report

## Abstract

**Background:**

Extra-gastric (particularly colonic) lymphoma of mucosa-associated lymphoid tissue in the immunosuppressed solid organ transplant recipient is rare. We report a case of low-volume mucosa-associated lymphoid tissue lymphoma with colonic and bone marrow involvement in a renal transplant recipient that has been managed conservatively.

**Case presentation:**

A 62-year-old Caucasian man, 14 years after kidney transplantation, was diagnosed as having extra-nodal marginal zone lymphoma of mucosa-associated lymphoid tissue with bone marrow and colonic involvement, after a colonoscopy identified mucosa-associated lymphoid tissue lymphoma in a sessile sigmoid polyp following surveillance fecal occult blood testing that returned a positive result. A gastric biopsy showed no evidence of *Helicobacter pylori*, but *Helicobacter pylori* immunoglobulin G was positive. He received *Helicobacter pylori* eradication treatment and is being managed expectantly. Immunosuppression was unchanged with prednisolone, mycophenolate mofetil, and cyclosporine A. Renal allograft function has remained stable.

**Conclusions:**

This case highlights the unexpected occurrence of colonic mucosa-associated lymphoid tissue lymphoma in a kidney transplant recipient. The case emphasizes the importance of histopathological diagnosis of colonic lesions in this patient cohort because the unusual diagnosis of low-volume mucosa-associated lymphoid tissue lymphoma can be managed expectantly as it does not appear to be clinically aggressive in the immunosuppressed solid organ transplant.

## Background

Colon cancer is three times more common in kidney transplant recipients compared to the general population [1]. Regular screening can reduce the outcome [2]. Mucosa-associated lymphoid tissue (MALT) lymphoma is an extra-nodal subtype of marginal zone lymphoma (a non-Hodgkin lymphoma) and is most commonly found in the gastrointestinal tract, particularly the stomach, with colonic involvement in only 2.5% of cases [3]. We report a case of a renal transplant recipient with an unexpected diagnosis of MALT lymphoma with colonic and bone marrow involvement; the diagnosis was made after a colonoscopy, following a positive bowel cancer surveillance test, identified MALT lymphoma in a sessile sigmoid polyp. Our patient was subsequently managed expectantly. To the best of our knowledge, there are only a few case reports of extra-gastric MALT lymphoma in solid organ transplant recipients, actively managed with a range of surgical, chemoradiotherapy, and immunotherapy treatments. This is the only reported case of low-volume MALT lymphoma with colonic and bone marrow involvement that has been managed expectantly with no clinical progression of the disease and stable allograft function 2 years after his initial MALT lymphoma diagnosis.

## Case presentation

This report describes an asymptomatic 62-year-old Caucasian man with a deceased-donor kidney transplant for end-stage kidney disease for obstructive uropathy due to renal calculi from childhood, 14 years post-transplantation, who was diagnosed as having extra-nodal marginal zone MALT lymphoma with bone marrow and colonic involvement; the diagnosis was made after a positive occult blood testing, which was conducted as part of routine cancer screening post-renal transplantation. He did not have any B symptoms including night sweats or unintended weight loss. He did not have any altered bowel habit and no overt per rectal bleeding. He did not have any proceeding infective symptoms prior to diagnosis.

His past medical history included gout, secondary hyperparathyroidism, osteoporosis, and a left lower limb deep venous thrombosis (previously on warfarin). He did not have any co-existing autoimmune conditions. Maintenance immunosuppression was cyclosporine A, mycophenolate mofetil, and prednisolone. Other medications were allopurinol, diltiazem, metoprolol, irbesartan, calcitriol, and cholecalciferol. He had no family history of colorectal carcinoma, inflammatory bowel disease, or coeliac disease. He lived at home with his wife, was independent and employed as a law lecturer. He never smoked tobacco and drank alcohol minimally.

On physical examination, he had an easily palpable transplant kidney but no other remarkable findings including no clinical lymphadenopathy. He had a baseline serum creatinine of 170 μmol/L and urine albumin-creatinine ratio of 4.7 mg/mmol after renal transplantation. He had a mild normocytic anemia with hemoglobin of 114 g/L. His other baseline laboratory results including liver function tests were unremarkable.

**Fig. 1:**
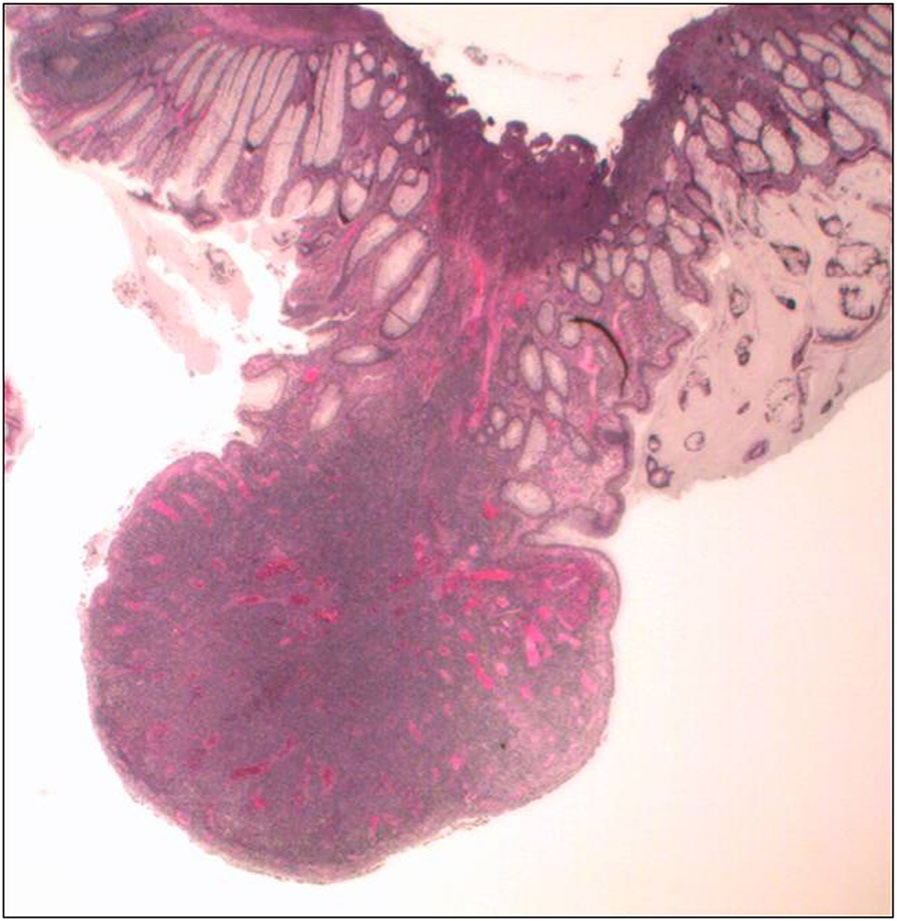
Sigmoid colon polyp. The mucosal polyp is composed of a monotonous infiltrate of small to medium sized lymphoid cells. The surface epithelium is ulcerated. Haematoxylin and eosin stain. Original magnification x20

**Fig. 2:**
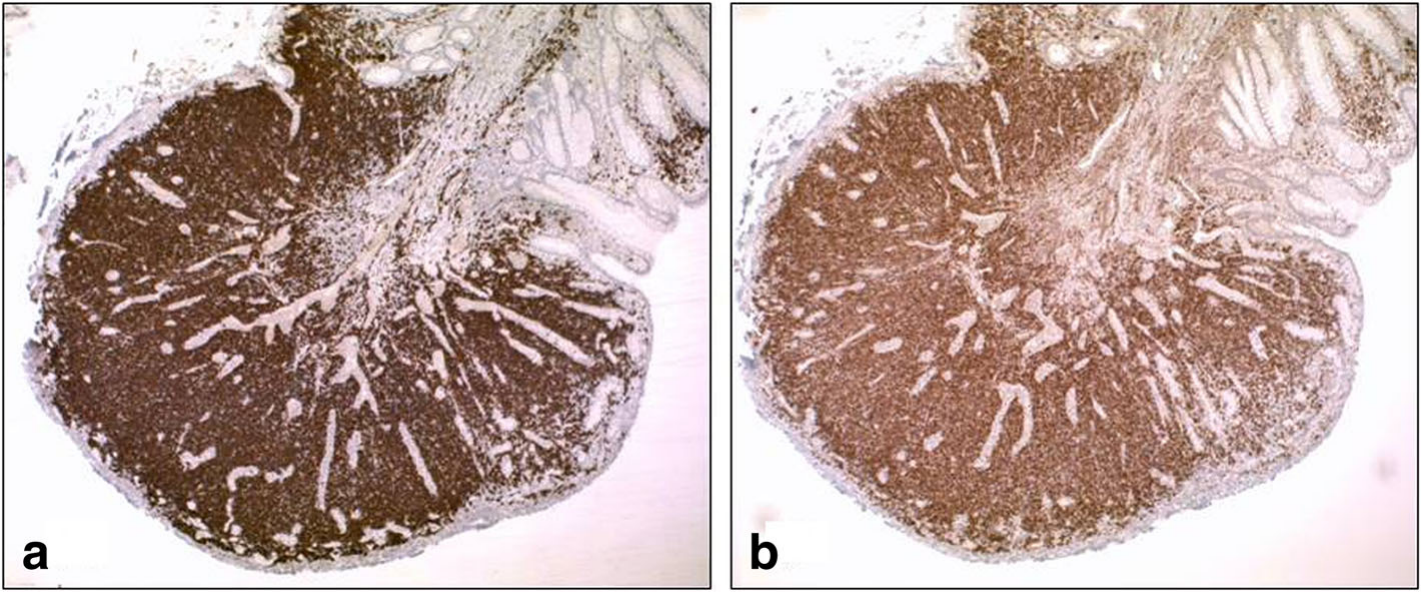
**a**. CD20 immunostain. The lymphoid infiltrate is composed of CD20-positive B-cells. **b**. BCL2 immunostain. The lymphoid cells show diffuse positive staining for BCL2 immunostain. Immunoperoxidase stain. Original magnification x40

Fecal occult blood testing was conducted as part of routine cancer surveillance, in the context of mild anemia, post kidney transplantation. Colonoscopy following a positive fecal occult blood test identified MALT lymphoma in one of three sessile sigmoid polyps (Figs. 1 and 2). The colonoscopy (inserted to the distal ileum) was otherwise normal macroscopically. Immunohistochemical (IHC) staining of the biopsy in the involved polyp showed a lymphoid population to be composed predominantly of small to medium-sized CD20-positive B cells. The cells were positive for BCL2 with negative staining for BCL6, CD10, cyclin D1, and CD5; Ki-67 proliferative index was 30–40%. Staining of light chains was suggestive of kappa restriction. Epstein–Barr encoding region *in situ* hybridization (EBERish) was negative. The other two polyps showed tubular adenomas with low-grade dysplasia. A gastroscopy (inserted to the duodenum) showed a patchy darker mucosa in the antrum and a 12 mm slightly raised circular patch with indistinct mucosal pattern in the mid-antrum. There was also a 10 mm indistinct slightly raised patch in the medial wall of distal D1 but it was otherwise normal macroscopically to D2. A gastric biopsy showed mild chronic gastritis with focal intestinal metaplasia and no evidence of *Helicobacter pylori* (*H. pylori*) or gastric lymphoma. The histopathology of the D1 and D2 biopsies were normal. Serum *H. pylori* IgG was positive. He received *H. pylori* eradication with esomeprazole, amoxicillin, and clarithromycin.

A further laboratory test was conducted to investigate for any systemic features of MALT lymphoma. A monoclonal IgA (kappa) protein was detected in the serum. Serum β2-microglobulin was raised, it was 5 mg/L, reference interval (RI) 0.8–3.0 mg/L, with normal lactate dehydrogenase of 176 U/L (RI < 250 U/L). His serum anti-nuclear antibodies and extractable nuclear antigen antibodies were negative. A bone marrow aspirate showed that 12% of nucleated cells were lymphocytes with a population of abnormal cells with a prominent nucleolus. Of the lymphocytes present, 50% were B cells of the phenotype CD5− CD10− CD20+ CD19+ CD22+ CD38− FMC7+ CD23− surface Ig+, kappa light chain. Monoclonality was demonstrated by light chain restriction. Immunoglobulin heavy chain (IgH) clonal rearrangement analysis demonstrated a monoclonal result. Bone marrow trephine histology showed only scattered CD20+ cells (20% of nucleated cells) with no lymphoid aggregates (not illustrated). Circulating lymphoma cells with the same immunophenotype as the bone marrow aspirate were detected in the peripheral blood. A positron emission tomography scan was negative for other disease sites.

Overall, these results were consistent with low-volume MALT lymphoma with bone marrow and colonic involvement. Our patient was asymptomatic and had no evidence of significant systemic disease. He was managed expectantly with monitoring of blood counts and paraprotein levels and repeat endoscopy. Immunosuppression was unchanged. Since the initial diagnosis, he has remained clinically well with stable renal allograft function after 2 years.

## Discussion

We describe a case of low-volume MALT lymphoma with colonic and bone marrow involvement in a 62-year-old man, 14 years after kidney transplantation. The diagnosis arose unexpectedly when a colonoscopy, following a positive surveillance fecal occult blood test result, identified MALT lymphoma in a polyp. There is no standardized therapy for extra-gastric MALT lymphoma and only a small case series published on the management of MALT lymphoma in solid organ transplant recipients. Unlike more aggressive therapies for management of MALT lymphoma in solid organ transplant recipients described in other literature, our patient has been managed conservatively with no change to immunosuppression. There has been no evidence of clinical progression after 2 years. This report highlights consideration of conservative management with regular clinical assessment in low-volume extra-gastric MALT lymphoma.

Colon cancer is the second most common solid organ cancer in renal transplant recipients and occurs three times more frequently in kidney transplant recipients compared to the general population [1]. In addition to age-appropriate cancer screening, fecal occult blood testing and colonoscopy can reduce the outcome [2].

Post-transplant lymphoproliferative disorders (PTLD) are commonly non-Hodgkin lymphomas and Epstein–Barr virus (EBV)-driven. The outcome is poor with survival at 1 and 5 years of 51% and 31%, respectively [4]. In contrast, MALT lymphoma is not classified as a PTLD. It comprises 7–8% of all B cell lymphomas, is usually low grade and localized to extra-nodal sites. It is not associated with EBV [5].

MALT lymphoma is most commonly found in the gastrointestinal tract, particularly the stomach, with colonic involvement in only 2.5% of cases [3]. One third of MALT lymphoma cases disseminate to other MALT sites, lymph nodes, or bone marrow. Complete remission occurs in 70–80% of cases of localized gastric MALT lymphoma after antibiotic treatment of *H. pylori* infection; however, this association is not seen in extra-gastric MALT lymphoma [6]. Involvement of other MALT sites, including bone marrow involvement, does not appear to confer a worse prognosis in previous reports [7].

MALT lymphoma is rare in a solid organ transplant recipient with only a small case series published [8]. It appears to present similarly and behave like MALT lymphoma in the immunocompetent patient and not like PTLD in the immunosuppressed solid organ transplant recipient [8]. There is no standardized therapy for extra-gastric sites; treatment includes surgical resection, radiotherapy, chemotherapy, and immunotherapy with anti-CD20 monoclonal antibodies such as rituximab [9]. Our patient was managed conservatively without active treatment of the low-grade MALT lymphoma. He has continued to be clinically well 2 years post initial diagnosis with no progression of the disease and stable renal allograft function.

## Conclusions

This case of unexpected colonic MALT lymphoma highlights the importance of judicious cancer screening with histopathological diagnosis of suspicious colonic lesions in immunosuppressed solid organ transplant recipients. Conservative management can be considered in extra-gastric MALT lymphoma in immunosuppressed solid organ transplant recipients with regular assessment of clinical progression as it does not appear to be clinically aggressive if the lymphoma is of low volume.

## Data Availability

Not applicable.

## Abbreviations

MALT: Mucosa-associated lymphoid tissue
IHC: Immunohistochemical
EBERish: Epstein–Barr encoding region *in situ* hybridization
RI: Reference interval
*H. pylori*: *Helicobacter pylori*
IgH: Immunoglobulin heavy chain
PTLD: Post-transplant lymphoproliferative disorders
EBV: Epstein–Barr virus
